# Structural Insights into a Novel Interkingdom Signaling Circuit by Cartography of the Ligand-Binding Sites of the Homologous Quorum Sensing LuxR-Family

**DOI:** 10.3390/ijms141020578

**Published:** 2013-10-15

**Authors:** Sonia Covaceuszach, Giuliano Degrassi, Vittorio Venturi, Doriano Lamba

**Affiliations:** 1 Institute of Crystallography, National Research Council, Trieste Outstation, Area Science Park-Basovizza, S.S. n° 14 Km 163.5, I-34149 Trieste, Italy; E-Mail: sonia.covaceuszach@ts.ic.cnr.it; 2 International Centre for Genetic Engineering and Biotechnology, Padriciano 99, I-34149 Trieste, Italy; E-Mail: degrassi@icgeb.org; 3 IBIOBA-CONICET-ICGEB, International Centre for Genetic Engineering and Biotechnology, Scientific and Technological Center, Godoy Cruz 2390, C1425FQD, Buenos Aires, Argentina

**Keywords:** quorum sensing, bacterial-plant communication, interkingdom signaling, Plant Associated Bacteria LuxR solos, ligand binding site, molecular modeling, structure-based multiple alignment

## Abstract

Recent studies have identified a novel interkingdom signaling circuit, via plant signaling molecules, and a bacterial sub-family of LuxR proteins, bridging eukaryotes and prokaryotes. Indeed pivotal plant-bacteria interactions are regulated by the so called Plant Associated Bacteria (PAB) LuxR solo regulators that, although closely related to the quorum sensing (QS) LuxR family, do not bind or respond to canonical quorum sensing *N*-acyl homoserine lactones (AHLs), but only to specific host plant signal molecules. The large body of structural data available for several members of the QS LuxR family complexed with different classes of ligands (AHLs and other compounds), has been exploited to dissect the cartography of their regulatory domains through structure-based multiple sequence alignments, structural superimposition and a comparative analysis of the contact residues involved in ligand binding. In the absence of experimentally determined structures of members of the PAB LuxR solos subfamily, an homology model of its prototype OryR is presented, aiming to elucidate the architecture of its ligand-binding site. The obtained model, in combination with the cartography of the regulatory domains of the homologous QS LuxRs, provides novel insights into the 3D structure of its ligand-binding site and unveils the probable molecular determinants responsible for differences in selectivity towards specific host plant signal molecules, rather than to canonical QS compounds.

## 1. Introduction

Research over the last 15 years has evidenced that intercellular communication frequently occurs in bacteria regulating gene expression in a cell density-dependent signaling, referred to as “quorum sensing” (QS) [1]. Gram-negative bacteria most commonly use *N*-acyl homoserine lactones (AHLs) as signal molecules; AHLs are synthesized by LuxI-family proteins and at high concentration (*i.e.*, high cell density) they bind to a cognate LuxR-family regulatory protein, which consequently binds target gene promoters. There is a class of LuxR-family proteins having the typical modular structure of QS LuxRs, which do not possess a cognate LuxI AHL synthase; these LuxR proteins have been called orphans or solos [2,3]. A sub-family of LuxR solos of Plant Associated Bacteria (PAB) has recently been shown to be part of a novel interkingdom signaling circuit, involved in communication between plant with both pathogens and beneficial bacteria [4,5]. It is likely that this sub-family of LuxRs of this interkingdom signaling circuit shares structural and functional similarities with the archetypical LuxI/R QS systems [6]. These PAB LuxR solos bind and respond to plant signals and probably have undergone coevolution with the host plant.

Five members of this PAB LuxR solos subfamily have been studied: XccR of *Xanthomonas campestris* pv. campestris (Xcc), OryR of *Xanthomonas oryzae* pv. *oryzae* (Xoo), PsoR of *Pseudomonas fluorescens*, XagR of *Xanthomonas axonopodis* pv. *glycines* (Xag) and NesR of *Sinorhizobium meliloti* [2,7–11]. With the exception of NesR, all have been demonstrated to respond to as yet chemically uncharacterized low molecular weight signal molecules synthesized by the host plant, with the final outcome of regulating crucial aspects of plant-bacteria interactions. Namely, OryR of the rice vascular pathogen Xoo, is involved in virulence; it responds to plant signals since the protein is solubilized and activates the expression of the neighboring *pip* and of motility genes only in the presence of plant extracts [4,7,9]. XccR of the crucifer pathogen Xcc also responds to an as yet unidentified plant compound and regulates the neighboring *pip* gene; the presence of the plant extracts allows XccR to bind to the *pip* promoter *in vitro* [8]. XagR of the soybean pathogen Xag which causes bacterial leaf pustule on soybean (*Glycine max*) is also involved in virulence [11]. As for XccR in Xcc, XagR in Xag also activates *pip* transcription *in planta* and temporal studies have indicated that *pip* transcription increases gradually after infection, reaching its greatest activity after 72 h, before slowly decreasing. PsoR responds to plant compounds of different plant species playing a role in biocontrol by rhizospheric *Pseudomonas fluorescens* via the control of transcriptional regulation of various anti-microbial-related genes [10]. NesR of *Sinorhizobium meliloti* is important for survival under stress and utilization of various carbon sources; the response to plant compounds has not yet been addressed [2].

Nevertheless, being closely related to the QS LuxRs, this sub-family of PAB LuxR solos share the same overall protein architecture, comprising two functional domains. In particular, members of the QS LuxR family are mainly composed of an *N*-terminal ligand-binding domain (the regulatory domain) [12,13] and a *C*-terminal helix-turn-helix DNA-binding domain [14,15], joined together by a short linker region. In QS systems, a conformational change is induced upon binding of the regulatory domain to the cognate AHL, most commonly then allowing the recognition of specific promoter regions by the DNA-binding domain and leading to transcriptional activation [16,17]. Indeed binding to the AHL is responsible for stability, correct folding [16] and most commonly dimerization, which in turns stabilizes the transcription factor allowing DNA binding [18].

**Figure 1:**
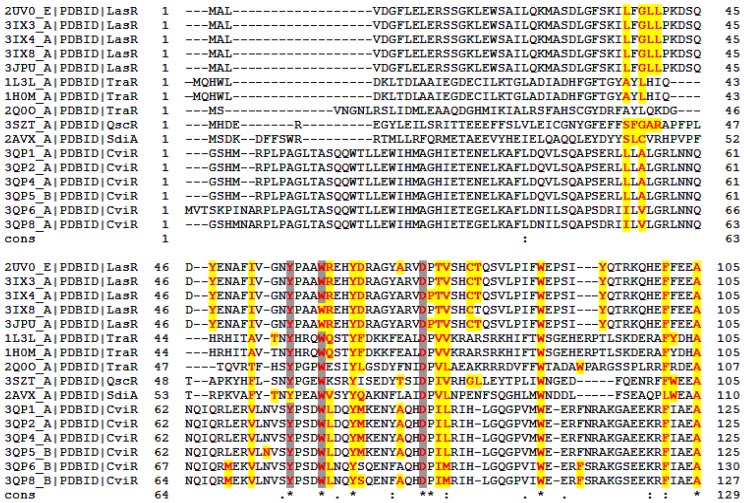

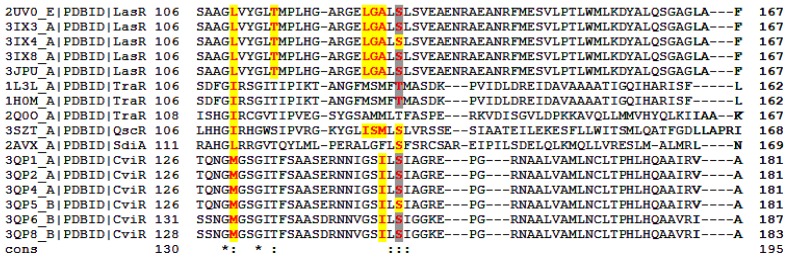
Structure-based multiple sequence alignment of the quorum sensing (QS) LuxRs whose structures have been solved to date. The residues directly involved in ligand binding are in bold and colored in red. The residues highlighted in yellow and in gray are engaged in hydrogen bonding and in hydrophobic interactions with the ligand, respectively. Stars highlight conserved positions; colon and period indicate respectively conservation between groups having strong or weak similarity. The unique identification PDB code (PDB_ID) of the 16 analyzed structures is given.

Surprisingly conservation of primary structure among LuxR-family proteins is quite low (18%–25%), however, multiple sequence alignments performed have identified nine highly conserved residues (Figure 1): six of these residues delineate the cavity of the ligand-binding domain (W57, Y61, D70, P71, W85 and G113, according to TraR numbering) and the remaining three are located within the DNA-binding domain (E178, L182 G188) [19–22]. On these bases in a recent review Gonzales and Venturi [4] pinpointed that members of PAB LuxR solos subfamily show substitutions in one or two of these highly conserved amino acids in the regulatory domain, namely, W57M and Y61W, thus suggesting an involvement of these residues in the different selectivity of this subfamily towards specific host plant signal molecules rather than to AHLs.

Considering that the experimental three dimensional structures of several members of the LuxR family [23–30] show a quite conserved overall folding, with the regulatory domain composed of five anti-parallel β-sheets flanked by three α-helixes on each side, our aim here is to: (i) validate and extend the previous analysis of LuxR-family, based on primary sequence alignment, in order to dissect the structural determinants involved in ligand recognition; and (ii) extend the outcomes of the detailed molecular cartography to the PAB LuxR solos subfamily in order to identify the molecular determinants responsible for the different ligand selectivity of this subfamily.

In the present study we take advantage of the large body of experimental structural data available for several members of the LuxR-family in complex not only with cognate AHLs, but also with unrelated signaling molecules [23–30], focusing on structure-based sequence alignment, structural superimposition and a comparative analysis of the contact residues involved in ligand binding; this should allow the identification of the key residues characterizing the ligand-binding sites. Moreover, in the absence of experimentally determined structures of members of the PAB LuxR solos subfamily, the homology model of its prototype, OryR, is expected to provide us with sufficient information to gain insights into the architecture of its ligand-binding site, as well as to elucidate the likely structural basis of the reported different ligand selectivity between the PAB LuxR solos subfamily and the canonical QS LuxR receptors.

## 2. Results and Discussion

Structure-based multiple sequence alignment of the regulatory domains of all the QS LuxRs whose experimental three-dimensional structural information (obtained by X-ray crystallography or by NMR spectroscopy) is available (*i.e.*, TraR from *Agrobacterium tumefaciens* and from *Sinorhizobium fredii* NGR234, LasR and QscR from *Pseudomonas aeruginosa*, CviR from *Chromobacterium violaceum* and SdiA from *Escherichia coli*) was performed by Expresso [31]. Figure 1 shows the multiple sequence alignment, based on structural information, having a main score (the total consistency value) of 71 (100 being the full agreement between the considered alignment and its associated primary library that has been computed as a first step of the consistency-based protocol exploited by Expresso), albeit the overall level of sequence identity or homology is quite low according to the calculated consensus sequence. It is interesting to note that even if the individual scores are 74 for LasR, 74 and 77 for the *A. tumefaciens* TraR and for its homolog from *S. fredii* NGR234 respectively, 76 for QscR, 56 for SdiA and 71 for CviR, regions encompassing residues 21–132 (TraR numbering) are characterized by an even higher level of consistency that has been prompted to reflect a higher level of accuracy [31].

The regulatory domains of all the QS LuxRs complexes in the PDB database [32] have been analyzed using Pymol [33] and the Protein Interfaces, Surfaces and Assemblies (PISA) interactive tool for the exploration of macromolecular—ligand interfaces at the European Bioinformatic Institute [34]. The results have been summarized in Figure 1 and will be discussed using TraR numbering as a reference.

It is interesting to note that most of the residues involved in ligand binding (see Figure 1, in bold and colored in red) are conserved in all the QS LuxRs complexes and seems to be invariant regardless of the chemical nature of the ligand (AHLs, chloro-lactones, triphenyl ligands) as observed in the LasR complexes. This finding supports the strategy to dissect the cartography of the ligand-binding sites of QS LuxRs in order to gain insight on the structural basis of PAB LuxR solos specificity.

Previous studies have suggested, based on multiple sequence alignment of QS LuxR transcriptional regulators, that six conserved hydrophobic/aromatic residues of the regulatory domain *i.e.*, W57, Y61, D70, P71, W85 and G113, delineate the binding site [19–22]. The present structure-based multiple sequence alignment validates the above mentioned six residues pinpoints to an additional three conserved residues of the QS LuxR family regulatory domain, *i.e.*, Y53, A105 and G109 (identified by a star in Figure 1) and clearly indicates that residues P71, G109 and G113, although located very close to the binding site, are not directly involved in ligand binding. Furthermore, an additional four, but not fully conserved, among the 10 residues with similar physico-chemical properties (identified by a semicolon in Figure 1) directly interact with the ligands in all the analyzed complexes.

Besides the 10 residues, *i.e.*, Y53, W57, Y61, D70, W85, A105; V/L73, F/L101, I/L/M110 and T/S129, in a number of QS LuxRs complexed with AHLs—*i.e.*, TraR from *A. tumefaciens* in complex with OC8-HLS (PDB_ID 1L3L [23], TraR from *S. fredii* NGR234 in complex with OC8-HSL (PDB_ID 2Q0O [25]); LasR from *P. aeruginosa* in complex with OC12-HSL (PDB_ID 2UV0 [26], PDB_ID 3IX3), QscR from *P. aeruginosa* in complex with OC12-HSL (PDB_ID 3SZT [28])—a water molecule is present at the ligand-binding sites, mediating protein-AHLs interactions by bridged hydrogen bonds.

**Figure 2:**
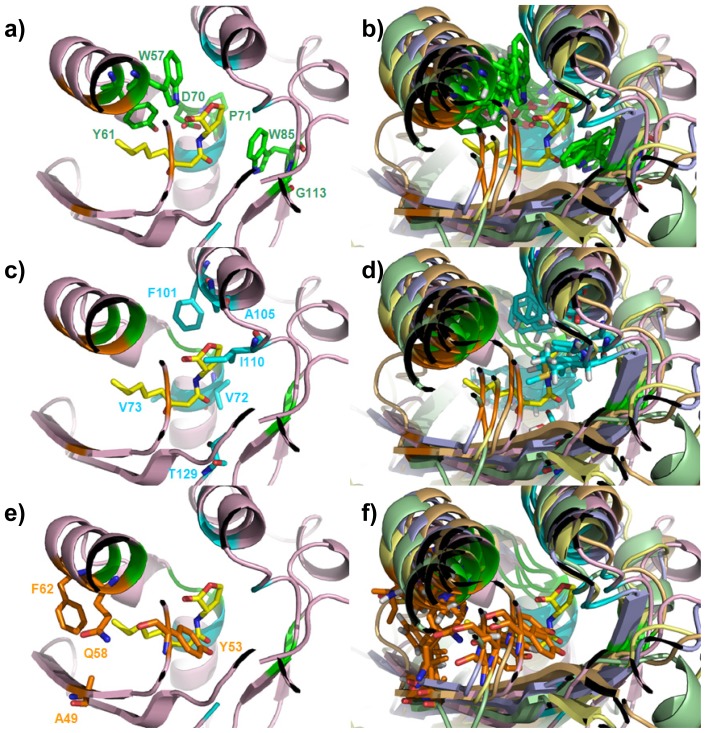
Mapping of the identified Clusters residues on the regulatory domains of the QS LuxRs. Residues, belonging to Cluster 1 (in green), Cluster 2 (in cyan) and Cluster 3 (in orange) and labeled according to the TraR numbering scheme, have been mapped in (**a**), (**c**) and (**e**) on the X-ray crystal structure of TraR in complex with OC8-HSL (PDB_ID 1H0M [24]); in (**b**), (**d**) and (**f**) on the superimposed C_α_ traces of the representative structures of the regulatory domains of QS LuxR, showing the side chains orientations, with TraR (PDB_ID 1H0M [24]) in light pink, LasR (PDB_ID 3IX3) in light orange, QscR (PDB_ID 3SZT [28]) in light yellow; SdiA (PDB_ID 2AVX [30]) in light blue, CviR (PDB_ID 3QP1 [29]) in light green. The carbon, nitrogen and oxygen atoms of OC8-HSL, are colored in yellow, blue and red respectively. Figures produced by Pymol [33].

From a structural perspective, it is interesting to note that not only the C_α_ positions but also the side chains orientations of all the six conserved residues W57, Y61, D70, P71, W85 and G113 (hereafter called Cluster 1, highlighted by a star in Figure 1 and colored in green in Figure 2) superimpose in all the analyzed structures rather well (Figure 2b). To this end only W57, Y61, D70 and W85 are directly involved in ligand binding (see Figures 1 and 2a). Residues P71 and G113 are located close to the binding site (Figure 2a) and are likely involved in the proper side chain orientation of D70 and W85, respectively. In this respect it is worth noting that in all of the analyzed structures, residues P71 and G113 adopt a *trans* and *cis* peptide conformation, respectively.

The present analysis reveals that the regulatory domain of the QS LuxR family includes, besides Cluster 1, an additional cluster of residues, namely V72, V73, F101, A105, I110, T129 (hereafter Cluster 2, colored in cyan in Figure 2) that is reasonably conserved and also directly involved in ligand binding (see Figures 1 and 2c). In all of the analyzed structures, the C_α_ positions and the side chain orientations superimpose rather well, as shown in Figure 2d.

**Figure 3:**
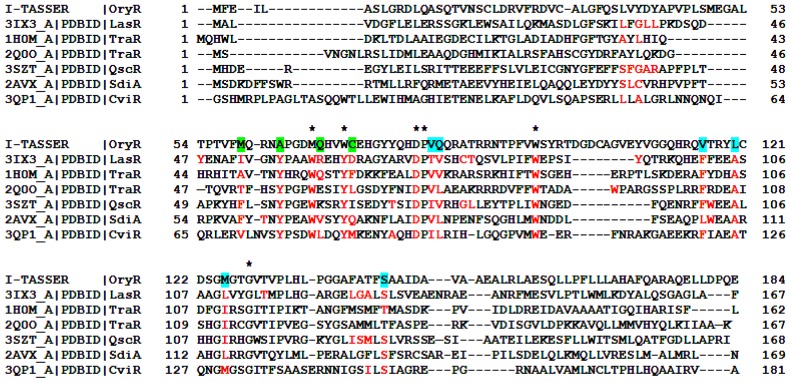
Structure-based multiple sequence alignment of the QS LuxRs with the prototype of the PAB LuxR solos subfamily. The residues of the QS LuxR involved in ligand binding are colored in red. The homologous residues of OryR, the prototype of PAB LuxR solos subfamily, belonging to Cluster 1 are highlighted by a star while the ones belonging to Cluster 2 and Cluster 3 (see text) are highlighted in cyan and in green respectively.

Beyond these two conserved clusters, the residues A49, Y53, Q58 and F62 (hereafter Cluster 3, colored in orange in Figure 2), represent a less conserved cluster (see Figures 1 and 2e) within the regulatory domain of the QS LuxR family. Besides Y53 that is conserved in a number of members belonging to the LuxR family (Figure 3), the residues A49, Q58 and F62 are highly substituted. Nevertheless the C_α_ positions and the side chains orientations of these residues superimpose rather well in all of the analyzed structures (Figure 2f).

To extend this detailed molecular cartography of the regulatory domain of the QS LuxR family to the PAB LuxR solos subfamily [35], OryR, the prototype of this subfamily, has been modeled and structurally aligned, based on secondary structure prediction, using I-TASSER [36] (see Figure 3).

The obtained homology model allowed to inspect the architecture of its ligand-binding site and to map the residues belonging to the three clusters, pinpointing the molecular determinants that are responsible for the observed differences in the ligand selectivity of this subfamily compared to QS LuxRs.

**Figure 4:**
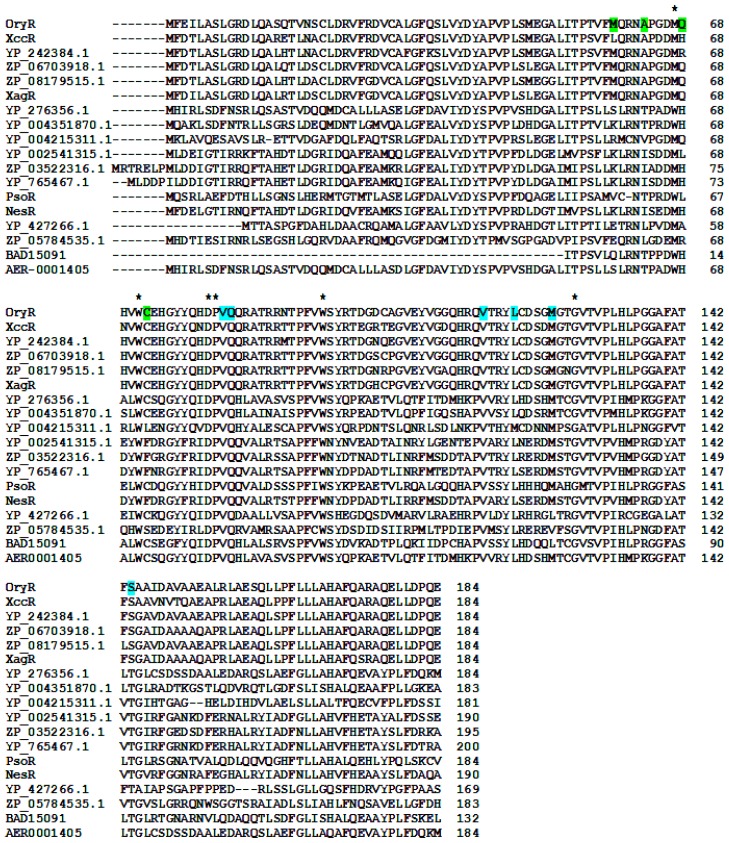
Multiple sequence alignment of the PAB LuxR solos subfamily members. The residues of the PAB LuxR solos subfamily homologous to Cluster 1 are highlighted by a star while the ones homologous to Cluster 2 and Cluster 3 (see text) are highlighted in cyan and in green, respectively.

Mapping Cluster 2 residues on the regulatory domain of OryR shows that residues V72 and T129 are conserved, whereas residue F101 is substituted by L (like in QscR) and I110 is substituted by M (similarly to CviR). V73 and A105 instead are substituted by Q and L respectively, these residues being rather conserved and specific for the subfamily of PAB LuxR solos (highlighted in cyan in Figures 3 and 4).

Regarding Cluster 3, the residues A49, Q58 and F62 are highly substituted in the PAB LuxR solos subfamily (highlighted in green in Figures 3 and 4) similar to what has been found in the QS LuxRs. In contrast, Y53 is highly variable within the PAB LuxR solos subfamily members (Figure 4), while it is conserved in a number of QS LuxRs (Figure 3).

Details of the residues type and the frequencies for the residues belonging to each of the three clusters both in QS LuxRs and in PAB LuxR solos are summarized in Table 1.

The three dimensional architecture of the boundaries of the ligand-binding site of the QS LuxRs is outlined in Table 2.

**Figure 5:**
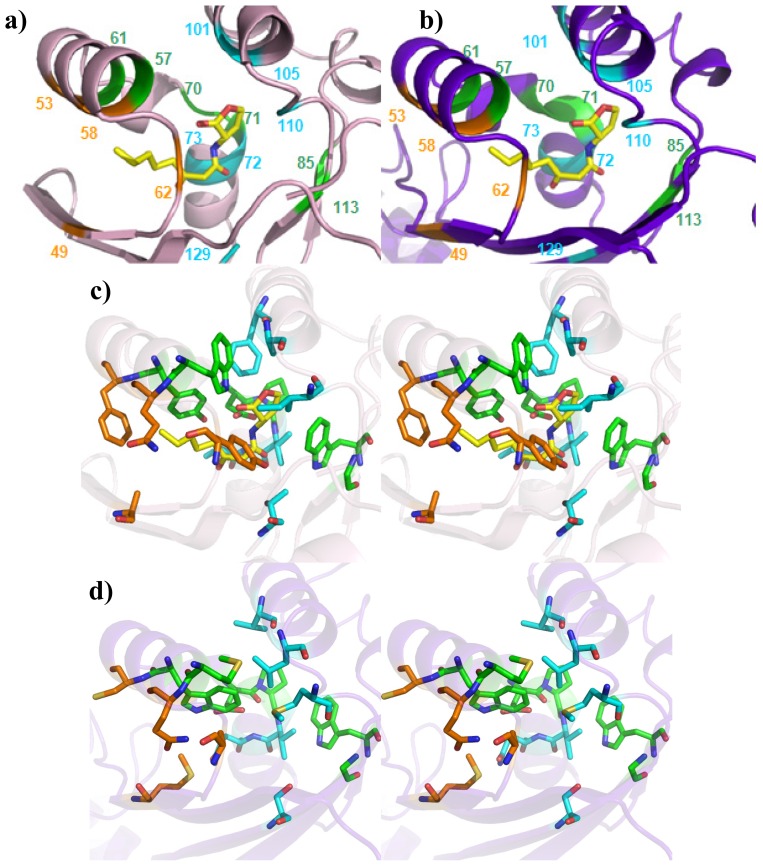
Comparison of the ligand-binding sites of prototypes of QS LuxRs and PAB LuxR solos subfamily (Top view). Mapping of the three Clusters of residues in (**a**) and (**c**) on the X-ray crystal structure of TraR, in complex with OC8-HSL (PDB_ID 1H0M [24]), showing the C_α_ trace and stereo view of the side chains, respectively; in (**b**) and (**d**) on the homology model of OryR, the C_α_ trace and stereo view of the side chains orientations can be seen. Residues have been labeled according to the TraR numbering scheme. The carbon, nitrogen and oxygen atoms of OC8-HSL, are colored in yellow, blue and red respectively. Figures produced by Pymol [33].

The contribution of the three clusters to the binding site topology of QS LuxRs and in PAB LuxR solos can be seen in Figure 5.

The residues belonging to each of the above described three clusters have been mapped on TraR (PDB_ID 1H0M) [24] and on the homology model of OryR regulatory domains (Figure 5a,b) in order to obtain the cartography of their respective ligand-binding sites: the resulting comparison (Figure 5c,d) indicates a tripartite architecture.

Firstly, a shared part (conserved core), delimited by the floor and the distal wall (residues 70, 71, 72, 85, 110, 113, 129), appears to be crucial for the folding mechanism of the regulatory domain in the presence of the signal molecule, regardless of its chemical nature. Then a specific part (specificity patch), mainly delimited by the roof and the nearby regions of the proximal and distal walls (residues 57, 61, 73, 101, 105), is conserved only within the QS LuxRs or within the PAB LuxR solos subfamily members respectively. It is therefore likely that the selectivity of LuxR family and of the PAB LuxR solos subfamily towards diverse ligands is modulated by these residues. In all the experimental structures analyzed, these are the ones interacting with the lactone ring of the AHL, while the PAB LuxR solos do not bind to AHLs. Finally a variable part (variability patch), delimited by the proximal wall and the nearby regions of the roof and of the floor (residues 49, 53, 58, 62), is less conserved even within the members of the QS LuxR family or of the PAB LuxR solos subfamily. It is interesting to note that in all the experimental structures analyzed, these residues interact with the fatty acyl side chain moieties of the AHLs that are found to adopt different position/orientation and conformations. Therefore they are likely to be responsible for the different selectivity towards molecules belonging to the same family of ligands or for the modulation of the degree “promiscuity” towards members of the same family of compounds.

**Figure 6:**
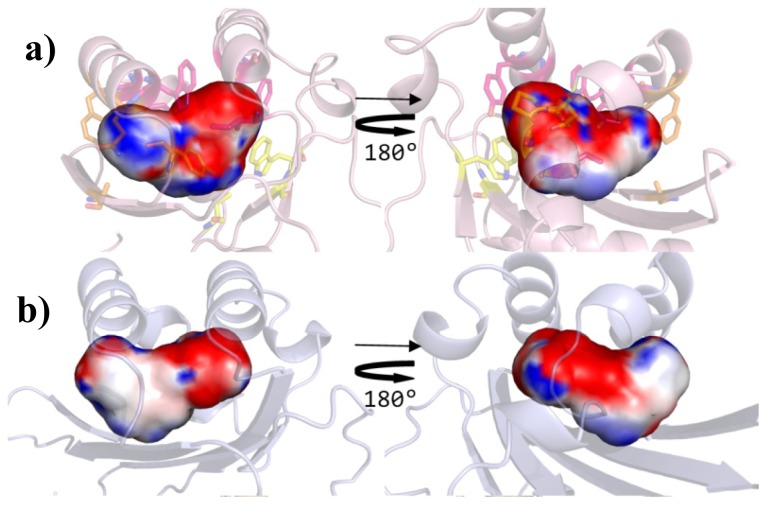

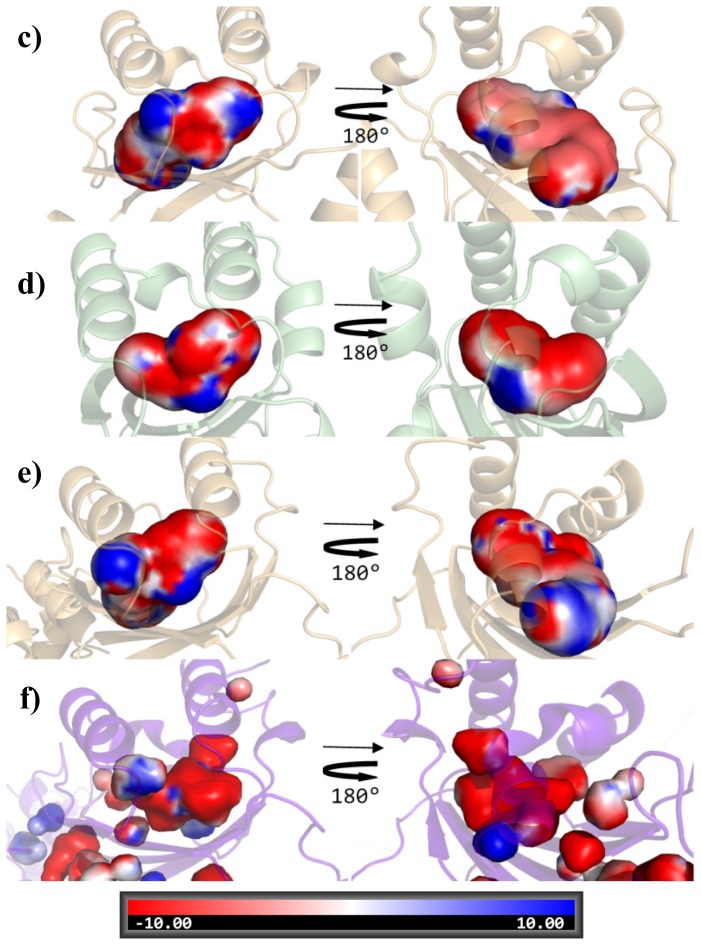
Electrostatic potential of the ligand-binding sites of the QS LuxR and of the prototype of the PAB LuxR solos subfamily. (**a**) TraR (PDB_ID 1H0M [24]) in light pink (showing the side chains of the conserved core in yellow, of the specificity patch in magenta and of the variable patch in orange); (**b**) SdiA (PDB_ID 2AVX [30]) in light blue; (**c**) LasR (PDB_ID 3IX3) in light orange; (**d**) CviR (PDB_ID 3QP1 [29]) in light green; (**e**) QscR (PDB_ID 3SZT [28]) in light yellow; (**f**) OryR model in blue purple. **Left** column: top view of the ligand binding sites (same orientation of the previous Figures); **Right** column: bottom view (obtained by 180 degrees rotation around *y* axis). Figures produced by Pymol [33].

**Figure 7:**
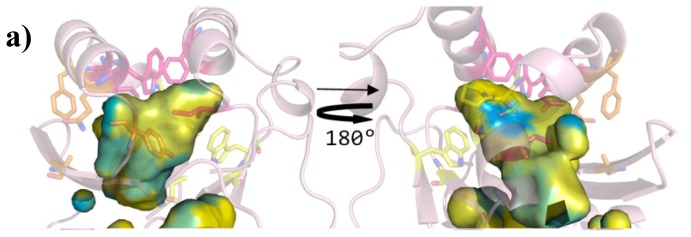

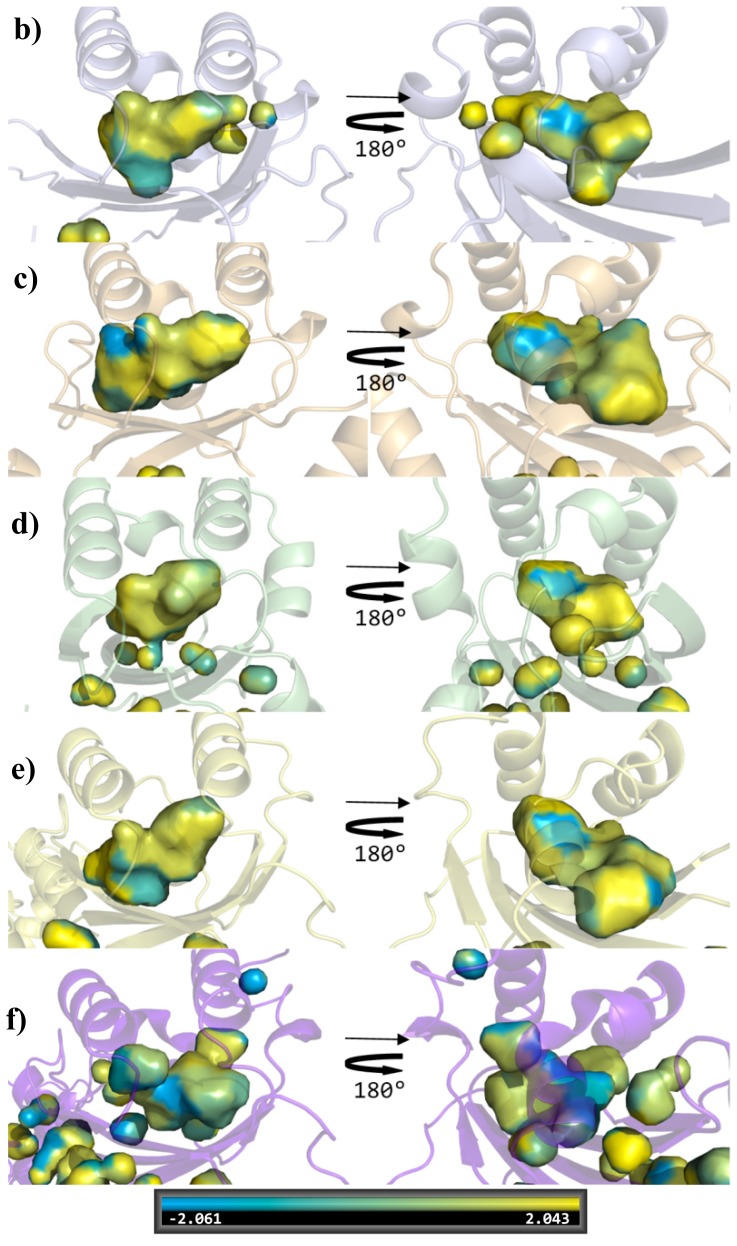
Lipophilic potential of the ligand-binding sites of the QS LuxR and of the prototype of the PAB LuxR solos subfamily. (**a**) TraR (PDB_ID 1H0M [24]) in light pink (showing the side chains of the conserved core in yellow, of the specificity patch in magenta and of the variable patch in orange); (**b**) SdiA (PDB_ID 2AVX [30]) in light blue; (**c**) LasR (PDB_ID 3IX3) in light orange; (**d**) CviR (PDB_ID 3QP1 [29]) in light green; (**e**) QscR (PDB_ID 3SZT [28]) in light yellow; (**f**) OryR model in blue purple. The hydrophobic surface is indicated by yellow and the hydrophilic surface by cyan. **Left** column: top view (same orientation of the previous Figures); **Right** column: bottom view (obtained by 180 degrees rotation around *y* axis). Figures produced by Pymol [33].

In order to corroborate the results of this analysis, the shape and the physico-chemical properties of the ligand-binding sites were evaluated. In Figures 6 and 7, on comparing the overall shape and the electrostatic and lipophilic potentials, respectively, of the ligand-binding sites of QS LuxRs, confirms the tripartite topology previously outlined (mapping the conserved core in yellow, the specificity patch in magenta and the variability patch in orange). Furthermore the physico-chemical properties mapped on the ligand-binding site of the OryR model reveal an increased negative potential (Figure 6) and a decreased hydrophobicity (Figure 7) in comparison to QS LuxRs, which most likely accounts for the structural determinants that are responsible for differences in the selectivity of the PAB LuxR solos subfamily with respect to QS LuxRs.

**Figure 8:**
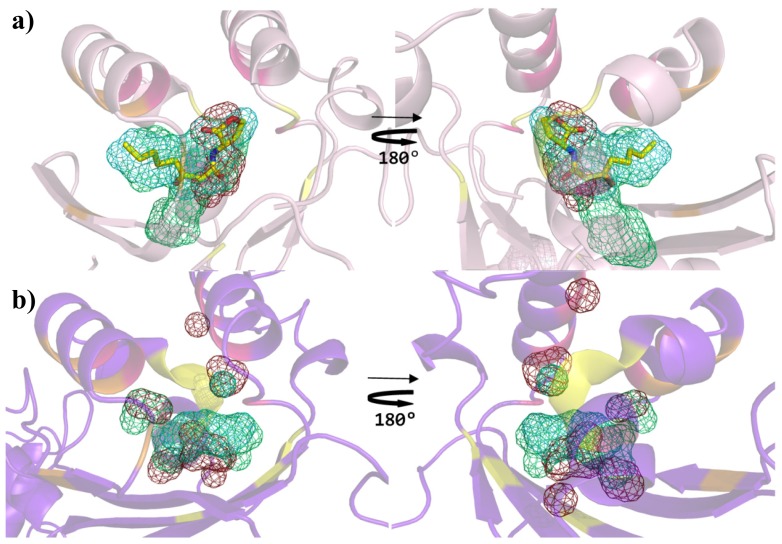
Comparison of the binding site preferences of prototypes of QS LuxRs and of PAB LuxR solos subfamily. Cartoon diagram (**a**) of TraR X-ray crystal structure in complex with OC8-HSL (PDB_ID 1H0M [24]) (showing the C_α_ trace of the conserved core in yellow, of the specificity patch in magenta and of the variable patch in orange) and (**b**) of the model of OryR, showing the top ranking clusters obtained by SITEHOUND [37] as mesh: methyl carbon probe clusters (green), aromatic carbon probe clusters (cyan) and hydroxyl oxygen probe cluster (red). The carbon, nitrogen and oxygen atoms of OC8-HSL are colored in yellow, blue and red respectively. Left column: top view of the ligand binding sites (same orientation as in previous figures); right column: bottom view (obtained by 180 degree rotation around *y* axis). Figures produced by Pymol [33].

An additional validation was carried out by comparing the binding sites topochemical preferences of the QS LuxRs and of the PAB LuxR solos prototypes by SITEHOUND [37] that exploits favorable interactions of three different structural probes (methyl carbon, aromatic carbon and hydroxyl oxygen). This analysis discloses clear differences between the prototypes of the two families (Figure 8). Indeed the comparison of the binding sites of the OryR and of the prototype of QS LuxRs shows the former to prefer hydroxylic groups rather than aliphatic and/or aromatic groups; therefore providing further support to the molecular determinants responsible for the differences in selectivity of the PAB LuxR solos towards specific host plant signal molecules rather than towards canonical quorum sensing ligands.

## 3. Experimental Section

Sequence alignment was performed by Expresso [31], that exploits structural aligners algorithms like SAP [38] or TMalign [39] to generate structure-based alignments that are used as a template for realigning the original sequences.

All the complexes of QS LuxR deposited in PDB both solved by X-ray crystallography, *i.e.*, TraR from *Agrobacterium tumefaciens* in complex with OC8-HSL (PDB_ID 1L3L [23], PDB_ID 1H0M [24]), TraR from *Sinorhizobium fredii* NGR234 in complex with OC8-HSL (PDB_ID 2Q0O [25]); LasR from *Pseudomonas aeruginosa* in complex with OC12-HSL (PDB_ID 2UV0 [26], PDB_ID 3IX3), with TP1 (PDB_ID 3IX4 [27]), with TP3 (PDB_ID 3IX8 [27]) and with TP4 (PDB_ID 3JPU [27]); QscR from *Pseudomonas aeruginosa* in complex with OC12-HSL (PDB_ID 3SZT [28]); CviR from *Chromobacterium violaceum* in complex with C6-HSL (PDB_ID 3QP1 and PDB_ID 3QP6 [29]), in complex with OC8-HSL (PDB_ID 3QP2 [29]), in complex with OC10-HSL (PDB_ID 3QP4 and PDB_ID 3QP8 [29]) and in complex with an antagonist chlorolactone (PDB_ID 3QP5 [29]) and by NMR, *i.e.*, SdiA from *Escherichia coli* in complex with OC8-HSL (PDB_ID 2AVX [30], have been superimposed using Pymol [33] and analyzed by using the Protein Interfaces, Surfaces and Assemblies (PISA) web tool at the European Bioinformatic Institute [34].

Homology-based protein modelling has been performed on the full-length amino acidic sequence of OryR protein from *Xanthomonas oryzae* using five molecular modelling strategies based on different criteria for template selection. SWISS-MODEL [40] performs a search in a library of experimental protein structures extracted from the PDB: up to five template structures per batch are superposed using an iterative least squares algorithm generating a structural alignment after removing incompatible templates, improved by a heuristic step after the calculation of a local pair-wise alignment of the target sequence to the main template structures [41]. ModWeb [42] depends on the large scale protein structure modeling pipeline, ModPipe, which performs a search in a set of non-redundant chains extracted from structures in the PDB and establishes sequence-structure matches using multiple variations of sequence-sequence, profile-sequence, sequence-profile and profile-profile alignment methods [43–45]. M4T (Multiple Mapping Method with Multiple Templates) [46] is based on two of major modules, Multiple Templates (MT) and Multiple Mapping Method (MMM) [47], developed to produce accurate alignments and models by minimizing the errors associated with the first two steps of modeling procedure (template recognition and alignment). HHpred (Homology detection & structure prediction by HMM-HMM comparison) [48] implements pairwise comparison of profile hidden Markov models (HMMs) to generate pairwise query-template alignments or multiple alignments of the query with a set of templates selected from the search results. I-TASSER (iterative threading assembly refinement) [36] generates three-dimensional atomic models from multiple threading alignments and iterative structural assembly simulations.

The five top-scored models generated have been ranked and validated by two protein model quality predictors ProQ [49] and AIDE [50], that have different and often complementary ability to properly assess the quality of protein structures and therefore their combined use can increase the reliability in the evaluation of model quality. The resulting outputs were consistent, pinpointing the top-scored model (confidence score 0.64) produced by I-TASSER, based on the QscR template (PDB_ID 3SZT [28]), as the most reliable candidate. Indeed the correctness of the selected model was confirmed by ProQ [49] (having Predicted LGscore of 4.299 and Predicted MaxSub of 0.437) and its overall best quality was validated by AIDE [50] (with a Predicted TM-score of 0.69 and a Predicted RMSD of 6.97).

Electrostatic potentials calculations were performed by PDB2PQR [51] and visualized by Pymol [33] on the ligand surface in the ligand-binding sites of the QS LuxRs and on the cavity surface of the ligand-binding site of OryR model. Lipophilic potential representation, based on the hydrophobicity scale derived by Black and Mould [52], was performed by Pymol [33] on cavity surface of the ligand-binding site of the QS LuxRs and of OryR model.

Binding sites preferences of QS LuxRs and of PAB LuxR solos prototypes, TraR and OryR respectively, were analyzed by SITEHOUND [37] employing three different structural probes (*i.e.*, methyl carbon, aromatic carbon and hydroxyl oxygen).

## 4. Conclusions

The present study was aimed to dissect the cartography of the ligand-binding sites of the QS LuxRs by structure-based sequence alignment, structural superimposition and comparative mapping residues directly involved in ligand binding. The structure-based analysis pinpointed the key residues crucial for ligand recognition and led us to identify a tripartite architecture of the ligand-binding sites that may account for differences in selectivity within the QS LuxR family. In order to extend this detailed molecular cartography of the regulatory domain to the PAB LuxR solos subfamily, an homology model of its prototype OryR has been obtained. Its comparative structural analysis allowed the identification of the key molecular determinants shaping the physico-chemical properties (electrostatic and lipophilic potentials). The resulting binding sites topochemical preferences are likely to be responsible for the difference in selectivity of the PAB LuxR solos towards specific host-plant signal molecules rather than towards canonical QS ligands.

## Figures and Tables

**Table 1:** Clusters residue type and frequency in the ligand-binding site of the regulatory domain of QS LuxRs and of the PAB LuxR solos subfamily.

Cluster	Position	QS LuxRs: residue and frequency	QS LuxRs: ligand binding frequency	PAB LuxR solos: residue and frequency
1	57	W (6/6)	16/16	M (13/18), W (5/18)
1	61	Y (6/6)	16/16	W (18/18)
1	70	D (6/6)	2/16	D (18/18)
1	71	P (6/6)	16/16	P (18/18)
1	85	W (6/6)	0/16	W (18/18)
1	113	G (6/6)	16/16	G (18/18)
2	72	V (3/6), I (2/6), T (1/6)	16/16	V (18/18)
2	73	V (3/6), L (3/6)	16/16	Q (18/18)
2	101	F (5/6), L (1/6)	16/16	V (18/18)
2	105	A (6/6)	16/16	L (17/18), M (1/18)
2	110	I (3/6), L (2/6), M (1/6)	16/16	M (15/18), L (2/18), V (1/18)
2	129	S (4/6), T (2/6)	16/16	S (6/18), T (12/18)
3	49	F (2/6), I (1/6), V (1/6), T (1/6), A (1/6)	16/16	M (5/18), K (5/18), E (2/18), S (2/18), L (1/18), V (1/18), Q (1/18), R (1/18)
3	53	Y (6/6)	16/16	A (6/18), T (5/18), I (4/18), L (2/18), V (1/18)
3	58	E (1/6), K (1/6), Q (1/6), R (1/6), L (1/6), V (1/6)	14/16	H (8/18), Q (5/18), R (2/18), L (2/18), A (1/18)
3	62	M (1/6), F (1/6), Q (1/6), D(1/6), L (1/6), I (1/6)	14/16	C (12/18), F (4/18), S (1/18), L (1/18)

**Table 2:** Clusters three-dimensional spatial distribution in the ligand-binding site of the regulatory domain of the QS LuxRs.

	Roof	Distal Wall	Floor	Proximal Wall
Structural determinants	Two anti-parallel α-helices	Two perpendicular α-helices fragments	Four anti-parallel β-strands	Loops
Cluster 1	W57, Y61	D70, P71	W85, G113	-
Cluster 2	F101, A105	V72, V73	T129	I110
Cluster 3	Q58, F62	-	A49	Y53
